# Evaluating the Lack of Association Between Preoperative Hormone Replacement Therapy and Increased Venous Thromboembolism After Arthroscopic Orthopedic Surgery

**DOI:** 10.7759/cureus.110726

**Published:** 2026-06-12

**Authors:** Ronak J Mahatme, Shawn A Moore, Priyanka Parameswaran, Anish Gangavaram, Nishanth Muthusamy, Samuel K Gerak, David L Bernholt, Brian M Grawe

**Affiliations:** 1 Department of Orthopedics, University of Cincinnati College of Medicine, Cincinnati, USA; 2 Department of Orthopedic Surgery, Cleveland Clinic Foundation, Cleveland, USA; 3 Department of Orthopedic Surgery, Division of Sports Medicine, University of Cincinnati College of Medicine, Cincinnati, USA

**Keywords:** arthroscopy, hormone replacement therapy, pulmonary embolism, venous thromboembolism, venous thrombosis

## Abstract

Background: Hormone replacement therapy (HRT) has been associated with increased venous thromboembolism (VTE) risk in select patient populations, raising concern regarding its perioperative safety in orthopedic surgery. However, the impact of preoperative HRT use on postoperative complications following common arthroscopic procedures remains poorly characterized.

Methods: The TriNetX Research Network was queried to identify adults aged 40-75 who underwent arthroscopy of the shoulder, elbow, hip, knee, or ankle. Patients with documented HRT use within three months prior to surgery were propensity-matched 1:1 to controls with no HRT use any time before or within three months of surgery. Exclusions included prior VTE events, coagulation disorders, active neoplasm, and kidney failure. Propensity matching accounted for age, sex, body mass index (BMI) (>30), diabetes, hypertension, hyperlipidemia, tobacco use, cardiovascular comorbidities, autoimmune disease, and procedure type. The primary outcome was 90-day VTE, defined as deep vein thrombosis (DVT) or pulmonary embolism (PE); secondary outcomes included hospital readmission, surgical site infection, and hemarthrosis.

Results: After propensity score matching, each cohort included 3,868 patients. Ninety-day VTE occurred in 0.7% of HRT patients vs. 1.0% of controls (risk ratio, 0.68; 95% confidence interval, 0.42-1.10; p = 0.14). DVT (0.6% vs. 0.9%, p = 0.15), PE (0.3% vs. 0.3%; p = 1.00), readmission (p = 1.00), infection (p = 0.54), and hemarthrosis (p = 1.00) rates were also similar between the groups.

Conclusion: In this large, multicenter, propensity-matched cohort, preoperative HRT was not associated with a statistically significant increase in 90-day VTE or other postoperative complications following arthroscopic surgery. These findings should be interpreted as reassuring but hypothesis-generating evidence rather than definitive guidance regarding perioperative HRT management.

## Introduction

Hormone replacement therapy (HRT), including estrogen for women and testosterone for men, is commonly prescribed for perimenopausal symptoms or symptomatic hypogonadism in adults aged 40-75 [1,2]. Although some large-scale trends show declining prescription rates in recent decades [3-6], HRT use remains prevalent, with a recent systematic review reporting that HRT was used in 4.7% of postmenopausal women in 2020 [5]. Recent studies have linked oral HRT to an increased risk of venous thromboembolism (VTE) [7,8]. Accordingly, understanding the association between preoperative HRT exposure and postoperative complications may inform perioperative risk assessment in elective orthopedic sports procedures.

Both estrogen and testosterone have biologic effects on coagulation and vascular physiology that may alter perioperative thrombotic risk. Testosterone is associated with polycythemia, which can produce hyperviscosity and hypertension [9]; these changes have been linked to increased VTE risk in select clinical settings [10-15]. Estrogen modulates multiple components of the coagulation pathway and inflammatory cascades, and has been correlated clinically with elevated VTE incidence across various populations [16].

Most prior orthopedic investigations of hormone-associated thrombotic risk have focused on oral contraceptives in younger patients [17,18] or on HRT in the context of total joint arthroplasty [19,20]. In contrast, the impact of HRT on postoperative complications after elective arthroscopic sports procedures, whether in the shoulder, elbow, knee, hip, or ankle, has not been well characterized. Arthroscopy patients are typically lower risk for VTE than arthroplasty patients due to shorter operative times, less physiologic stress, and routine outpatient pathways [21,22]; thus, it is unclear whether the known mechanistic risks of HRT would lead to clinically meaningful increases in complications following these relatively low-risk arthroscopic procedures.

The purpose of this study was to evaluate whether preoperative HRT use is associated with 90-day postoperative VTE risk or other complications following common arthroscopic procedures. The primary outcome was 90-day VTE, defined as deep vein thrombosis (DVT) or pulmonary embolism (PE). Secondary outcomes included hospital readmission, postoperative infection, and postoperative hemarthrosis. We hypothesized that HRT use would be associated with increased postoperative VTE risk. Given the limited evidence regarding HRT use in arthroscopic populations, this study aimed to provide observational data to inform perioperative risk stratification and guide future procedure- and formulation-specific investigations.

## Materials and methods

This study was conducted in accordance with the ethical standards of the responsible committee on human experimentation (institutional and national) and with the Helsinki Declaration of 1975, as revised in 2008. The data used in this retrospective cohort study were collected from the TriNetX Research Network [23], which provided access to electronic medical records (diagnoses, procedures, medications, laboratory values, and genomic information) for over 150 million patients across 102 healthcare organizations. TriNetX, LLC (Cambridge, MA) [23] is compliant with the Health Insurance Portability and Accountability Act and applicable data privacy regulations, and all data accessed through the platform are deidentified in accordance with federal standards. Because this study used only deidentified patient records and did not involve the collection, use, or transmission of individually identifiable data, this study was exempt from Institutional Review Board approval.

Patients aged 40-75 years were included to encapsulate common populations treated with HRT for symptomatic hypogonadism or menopausal symptoms. This age range was selected to capture the population most commonly receiving menopausal HRT or testosterone replacement in the arthroscopic setting. HRT in our study is defined as conjugated estrogens (injectable or oral products), estradiol (drug implant, injectable, or oral products), or testosterone (drug implant, injectable, topical, or oral products). HRT exposure was analyzed as a pooled medication category because subgroup-level analyses by hormone type, formulation, dose, or route of administration were limited by the availability of TriNetX medication coding and low event counts. Therefore, this study evaluates documented preoperative HRT exposure broadly and does not determine the risk associated with specific estrogen or testosterone regimens.

Two cohorts were defined: 1) HRT use within three months prior to shoulder, elbow, knee, hip, or ankle arthroscopy; and 2) no HRT use at any time prior to and three months following arthroscopy. Patients were excluded if they had osteoporosis with acute pathological fractures, prior VTE, anemias, coagulation disorders, other blood disorders, neoplasms, kidney failure, or thrombophilia (Table 1). Patients with any documented HRT exposure outside the preoperative exposure window were excluded from the control group to reduce exposure misclassification.

**Table 1:** Inclusion/exclusion criteria HRT, hormone replacement therapy; VTE, venous thromboembolism

Category	Inclusion criteria	Exclusion criteria
Age	40-75 years	-
HRT exposure (cohort 1)	Documented HRT use within 3 months prior to arthroscopy	-
HRT exposure (cohort 2)	No HRT use at any time prior to or within 3 months following arthroscopy	-
Procedure	Arthroscopy of the shoulder, elbow, knee, hip, or ankle	-
Prior VTE	-	Prior venous thromboembolism
Bone/fracture	-	Osteoporosis with acute pathological fracture
Hematologic	-	Anemias, coagulation disorders, other blood disorders
Oncologic	-	Active neoplasm
Renal	-	Kidney failure
Hypercoagulable state	-	Thrombophilia

Propensity score matching was performed using a 1:1 greedy nearest neighbor algorithm without replacement. A caliper of 0.1 pooled standard deviations was utilized to ensure close covariate balance. Propensity scores were estimated using a multivariable logistic regression, including age, sex, BMI, type 2 diabetes mellitus, hypertension, hyperlipidemia, tobacco use, cardiovascular comorbidities, autoimmune disease, and anatomic location of arthroscopic procedure (Table 2). Standardized mean difference (SMDs) were used to assess covariate balance, with values <0.10 indicating appropriate balance. While propensity score matching reduces measured confounding, residual confounding may be present due to unmeasured variables. Only patients with complete data for all matching variables were included, and no imputation was performed. The proportion of excluded patients due to incomplete matching variables could not be fully characterized from the available aggregate TriNetX output.

**Table 2:** Propensity score matching for arthroscopic orthopedic procedures ACL, anterior cruciate ligament; BMI, body mass index; HRT, hormone replacement therapy; OCD, osteochondral defect; PCL, posterior cruciate ligament; SD, standard deviation; SMD, standardized mean difference

Variable	Before matching		SMD	After matching		SMD
	HRT (n = 4,169)	No HRT (n = 418,128)		HRT (n = 3,868)	No HRT (n = 3,868)	
Age, mean ± SD (years)	55.4 ± 8.1	55.0 ± 8.9	0.054	55.4 ± 8.1	55.4 ± 8.2	0.008
Female sex	2,005 (51.8%)	176,406 (43.7%)	0.162	2,002 (51.8%)	2,012 (52.0%)	0.005
Male sex	1,817 (46.9%	210,288 (52.1%)	0.104	1,815 (46.9%)	1,804 (46.6%)	0.006
BMI 30-39 kg/m²	449 (11.6%)	41,869 (10.4%)	0.039	448 (11.6%)	406 (10.5%)	0.035
BMI ≥40 kg/m²	153 (4.0%)	14,955 (3.7%)	0.013	153 (4.0%)	151 (3.9%)	0.003
Type 2 diabetes mellitus	519 (13.4%)	44,096 (10.9%)	0.076	519 (13.4%)	500 (12.9%)	0.015
Tobacco use	106 (2.7%)	8,844 (2.2%)	0.035	106 (2.7%)	99 (2.6%)	0.011
Hypertension	1,661 (42.9%)	132,624 (32.9%)	0.208	1,658 (42.9%)	1,640 (42.4%)	0.009
Hyperlipidemia	1,217 (31.4%)	90,330 (22.4%)	0.205	1,213 (31.4%)	1,218 (31.5%)	0.003
Acute myocardial infarction	27 (0.7%)	3,217 (0.8%)	0.012	27 (0.7%)	26 (0.7%)	0.003
Rheumatoid arthritis	75 (1.9%)	4,801 (1.2%)	0.060	74 (1.9%)	73 (1.9%)	0.002
Systemic lupus erythematosus	26 (0.7%)	1,240 (0.3%)	0.052	26 (0.7%)	20 (0.5%)	0.020
Heart failure	36 (0.9%)	3,393 (0.8%)	0.009	36 (0.9%)	37 (1.0%)	0.003
Atrial fibrillation	60 (1.5%)	4,965 (1.2%)	0.027	60 (1.6%)	54 (1.4%)	0.013
Osteoporosis without pathologic fracture	105 (2.7%)	8,268 (2.0%)	0.043	105 (2.7%)	93 (2.4%)	0.020
Shoulder arthroscopy, surgical	1,886 (48.7%)	174,764 (43.3%)	0.108	1,881 (48.6%)	1,866 (48.2%)	0.008
Elbow arthroscopy, surgical	29 (0.7%)	2,460 (0.6%)	0.017	26 (0.7%)	26 (0.7%)	<0.001
Hip arthroscopy, surgical	131 (3.4%)	9,897 (2.5%)	0.055	129 (3.3%)	141 (3.6%)	0.017
Knee arthroscopy, surgical	1,765 (45.6%)	196,145 (48.6%)	0.061	1,762 (45.6%)	1,762 (45.6%)	<0.001
Ankle arthroscopy, surgical	130 (3.4%)	11,776 (2.9%)	0.025	130 (3.4%)	131 (3.4%)	0.001
Diagnostic shoulder arthroscopy	14 (0.4%)	1,993 (0.5%)	0.020	14 (0.4%)	12 (0.3%)	0.009
Diagnostic elbow arthroscopy	10 (0.3%)	88 (0.0%)	0.063	10 (0.3%)	0 (0%)	0.072
Diagnostic hip arthroscopy	10 (0.3%)	209 (0.1%)	0.052	10 (0.3%)	10 (0.3%)	<0.001
Diagnostic knee arthroscopy	48 (1.2%)	2,525 (0.6%)	0.064	48 (1.2%)	56 (1.4%)	0.018
Arthroscopic ACL reconstruction	139 (3.6%)	19,128 (4.7%)	0.058	139 (3.6%)	139 (3.6%)	<0.001
Arthroscopic PCL reconstruction	10 (0.3%)	534 (0.1%)	0.029	10 (0.3%)	10 (0.3%)	<0.001
Arthroscopic ankle OCD excision/drilling	44 (1.1%)	3,298 (0.8%)	0.032	44 (1.1%)	44 (1.1%)	<0.001
Arthroscopic talar dome/plafond fixation	10 (0.3%)	589 (0.1%)	0.025	10 (0.3%)	10 (0.3%)	<0.001

The primary outcomes assessed within 90 days postoperatively included VTE, DVT, and PE. In addition, secondary outcomes analyzed at 90 days included hospital readmission, postoperative infection, and postoperative hemarthrosis. Outcomes with low event counts (≤10) were suppressed in accordance with TriNetX privacy policies.

Patient queries, demographic factors, and outcomes of interest were identified using Current Procedural Terminology (a standardized procedural coding system maintained by the American Medical Association), International Classification of Diseases, 10th Revision, Clinical Modification (a standardized diagnostic coding system maintained by the Centers for Disease Control and Prevention), and RxNorm (a standardized medication nomenclature developed by the National Library of Medicine) codes [24-26]. All codes used are provided in the Appendix.

Risk ratios (RRs) and 95% confidence intervals (CIs) were computed, and the complication rates were analyzed using the TriNetX system [23]. Categorical variables were assessed using the chi-squared test, while continuous variables were evaluated with independent t-tests. The level of statistical significance was set at p < 0.05. Because TriNetX suppresses event counts ≤10 to protect privacy, RRs and p values involving suppressed cells were interpreted with caution. For outcomes with suppressed event counts, statistical estimates may be imprecise and should be considered descriptive rather than definitive.

## Results

Prior to propensity score matching, 4,169 patients met the inclusion criteria for the HRT cohort, and 418,128 patients met the inclusion criteria for the control cohort. After propensity score matching for patients undergoing orthopedic arthroscopic procedures, the preoperative HRT cohort and the control cohort each had 3,868 patients. All covariates, including age, sex, BMI, type 2 diabetes mellitus, tobacco use, hypertension, hyperlipidemia, cardiovascular comorbidities, autoimmune disease, and anatomic location of arthroscopic procedure, were appropriately balanced as indicated by standardized mean differences (SMDs) <0.10 (Table 2).

**Figure 1:**
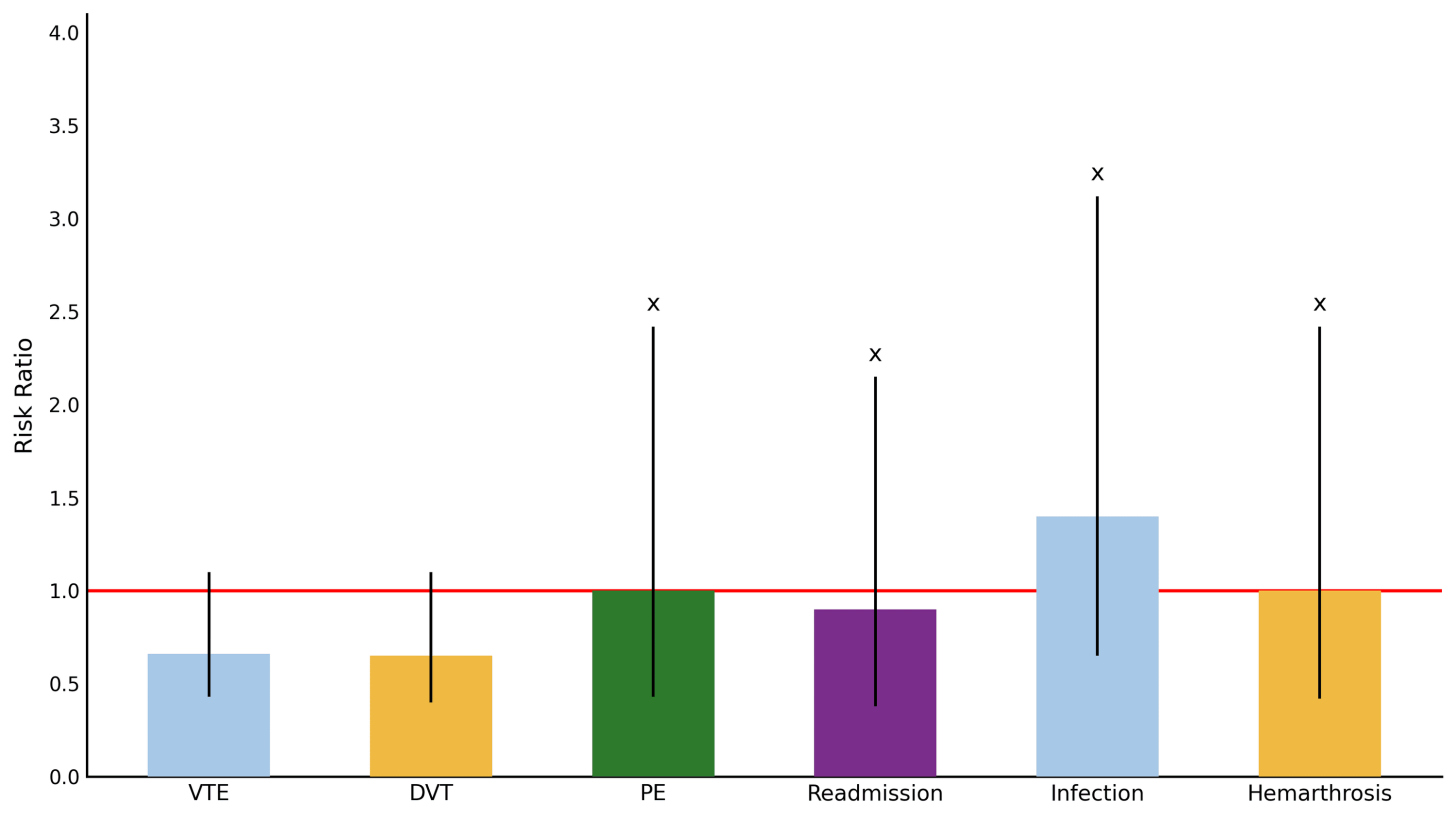
Ninety-day outcomes between HRT and control groups following orthopedic arthroscopic surgery X ≤10 patients in at least one group due to TrinetX protection of patient information VTE, venous thromboembolism; DVT, deep vein thrombosis; PE, pulmonary embolism; HRT, hormone replacement therapy

No statistically significant association was observed between preoperative HRT use and 90-day postoperative VTE. Specifically, the incidence of VTE in the HRT cohort (0.7%) was similar to the incidence of VTE in the control cohort (1.0%) (RR: 0.675; 95% CI: 0.415-1.098; p = 0.141). When VTE was further stratified, no statistically significant differences were observed in DVT or PE between the cohorts. Additionally, no statistically significant differences were observed in 90-day readmission rates, infection, or hemarthrosis between the two cohorts (Table 3, Figure 1).

**Table 3:** 90-day outcomes between preoperative HRT and control groups following orthopedic arthroscopic surgery ^*^Event counts are less than 11 and rounded due to TriNetX limitations in reporting small event count data to prevent patient deindividualization ^^^p values may not accurately represent significance as calculations are done with rounded patient populations VTE, venous thromboembolism; DVT, deep vein thrombosis; PE, pulmonary embolism; HRT, hormone replacement therapy; CI, confidence interval; df, degrees of freedom

Outcome	HRT events (%)	Control events (%)	Total patients per cohort	Risk ratio	95% CI	p value	χ² (df)
VTE	27 (0.7)	40 (1.0)	3,868	0.675	0.415-1.098	0.141	2.544 (1)
DVT	24 (0.6)	36 (0.9)	3,868	0.667	0.399-1.115	0.154	2.419 (1)
PE	≤10^*^ (0.3)	≤10^*^ (0.3)	3,868	1.000	0.417-2.400	1.000^^^	0.000 (1)
Readmission	≤10^*^ (0.3)	11 (0.3)	3,868	0.909	0.387-2.138	1.000^^^	0.048 (1)
Infection	14 (0.4)	≤10^*^ (0.3)	3,868	1.400	0.623-3.148	0.540^^^	0.669 (1)
Hemarthrosis	≤10^*^ (0.3)	≤10^*^ (0.3)	3,868	1.000	0.417-2.400	1.000^^^	0.000 (1)

## Discussion

To the best of our knowledge, this is the first large-scale database study to examine the association between preoperative HRT use and thromboembolic complications following arthroscopic surgery. The primary finding was that preoperative HRT use was not associated with a statistically significant increase in 90-day VTE, DVT, or PE compared with matched controls. Similarly, no statistically significant differences were observed in readmission, infection, or hemarthrosis. These findings should be interpreted as observational and hypothesis-generating, particularly given low event rates and heterogeneity in HRT type, formulation, and route of administration.

HRT use was not associated with a statistically significant increase in postoperative thromboembolic events in this matched arthroscopy cohort. Although point estimates for VTE and DVT were below 1.0, the CIs crossed the null and therefore should not be interpreted as evidence of a protective effect. Prior studies evaluating estrogen-containing therapies have demonstrated increased VTE risk in select patient populations [17,27,28]; however, literature regarding perioperative HRT continuation remains limited and occasionally contradictory [18]. Notably, some studies align with our observations and suggest that perioperative estrogen exposure may not be associated with increased VTE risk in select surgical populations [19,20]. Interpretation of the present findings remains complex because HRT encompasses biologically heterogeneous therapies, and thromboembolic effects may vary substantially according to hormone type, formulation, dose, and route of administration.

The effect of perioperative testosterone on VTE risk remains poorly characterized, though meta-analytic data suggest that testosterone does not increase VTE risk in the general population [29]. Biologically, testosterone increases hemoglobin and hematocrit [30], which can theoretically increase viscosity and thrombosis risk, but it also exerts vasodilatory and anti-inflammatory effects that may offset this risk [31]. These opposing mechanisms likely contribute to the inconsistent findings across the literature. While some studies have reported a transient rise in VTE risk shortly after testosterone initiation [32], multiple large-scale analyses have found no evidence that testosterone is a persistent or independent risk factor for thromboembolic events [29,33-35]. The low incidence of VTE observed in our matched arthroscopy cohort likely reflects both the minimal surgical stress of arthroscopic procedures and routine perioperative risk-mitigation practices. Although no statistically significant increase in VTE was observed in this cohort, these findings should not be interpreted as evidence that perioperative HRT is universally safe. Importantly, estrogen and testosterone may influence thrombosis through distinct mechanisms, and route of administration may further modify risk. Oral estrogen, transdermal estrogen, injectable testosterone, topical testosterone, and other formulations may not carry equivalent thromboembolic profiles. Because these therapies were analyzed together, the present findings should not be used to infer safety or risk for any specific hormone type, dose, or route.

Beyond thromboembolic outcomes, HRT use was not associated with higher rates of readmission, infection, or hemarthrosis. These results contrast with studies in arthroplasty populations, where HRT has been linked to increased risk of revision, periprosthetic joint infection, and pooled joint complications [36,37]. The relatively limited physiologic insult, lower baseline comorbidity burden, and routine outpatient nature of arthroscopic procedures likely mitigate these risks [38,39]. Given these factors, it is unsurprising that short-term outcomes, including readmissions and infections, were comparable between HRT and control patients. However, arthroplasty patients have substantially higher baseline thromboembolic and infectious risks than arthroscopy patients; therefore, comparisons between these populations should be interpreted with caution.

This study has several limitations. As with all retrospective database analyses, coding inaccuracies, exposure misclassification, residual confounding, and selection bias from unmeasured variables remain possible despite propensity score matching. Restricting inclusion to patients aged 40-75 years may limit generalizability to younger patients receiving testosterone replacement therapy. HRT was analyzed as a pooled exposure and could not be stratified by hormone type, formulation, dose, route of administration, or medication adherence. Because estrogen and testosterone have distinct biologic mechanisms and different formulations may confer variable thromboembolic risk, this study cannot determine the perioperative safety of any specific HRT regimen. Additionally, the control cohort excluded patients with prior documented HRT exposure, which may introduce selection bias if baseline health characteristics were not fully captured through matching. Complete-case analysis may also introduce bias if missing data were nonrandom. Furthermore, TriNetX suppression of event counts ≤10 further limits the interpretation of rare outcomes and may reduce power to detect small but clinically meaningful differences. Finally, perioperative anticoagulation practices, postoperative mobilization, operative time, surgeon-specific factors, and long-term outcomes were unavailable within the database and may influence postoperative risk. Despite these limitations, this study provides useful observational evidence from a large multicenter propensity-matched cohort. While the findings do not establish that perioperative HRT is risk-free or support recommendations regarding continuation or cessation of specific regimens, the absence of a statistically significant increase in short-term postoperative VTE suggests that a large increase in thromboembolic risk associated with pooled HRT exposure was not observed in this matched arthroscopic population. These findings provide preliminary observational evidence and support future procedure-specific and formulation-specific investigation of perioperative HRT use.

## Conclusions

In this large, multicenter, propensity-matched cohort, preoperative HRT use was not associated with a statistically significant increase in 90-day VTE or other short-term postoperative complications following arthroscopic orthopedic surgery. However, these findings should be interpreted with caution given the observational design, low event rates, a pooled HRT exposure definition, and the inability to distinguish among hormone type, dose, or route of administration. While these results provide preliminary reassurance, they should be considered hypothesis-generating and warrant further procedure- and formulation-specific investigation before firm perioperative HRT management recommendations can be made.

## Appendices

**Table 4:** CPT, ICD-10-CM, and RxNorm codes for TrinetX queries, propensity score matching, and outcomes VTE, venous thromboembolism; DVT, deep vein thrombosis; PE, pulmonary embolism; HRT, hormone replacement therapy; CPT, Current Procedural Terminology; ICD-10-CM, International Classification of Diseases, Tenth Revision, Clinical Modification

Diagnosis	Codes
Orthopedic arthroscopic surgery	1005614, 1005627, 1005641, 1005644, 1005648, 1005652, 1005657, 1005679, 29805, 29830, 29860, 29870, 29888, 29889, 29891, 29892
Osteoporosis with current pathological fracture	M80
Blood disorders and anticoagulant use	D55-D59, D60-D64, D65-D69, D70-D77, Z79.01, D68.5, D68.6
Kidney failure	N17-N19
Neoplasms	C40-C41, D45, D47, C81-C96
HRT	4083 (drug implant, injectable, or oral products), 4099 (injectable or oral products), 10379 (drug implant, injectable, topical, or oral products)
Propensity score inputs	Age at Index, Female, Male, E11, Z68.3, Z68.4, Z72.0, I10, E78.5, I21, M06.9, M32, I50, I48.91, M81, 1005614, 1005627, 1005641, 1005644, 1005648, 1005652, 1005657, 1005679, 29805, 29830, 29860, 29870, 29888, 29889, 29891, 29892
Postprocedural infection	T81.4
DVT	I82, T84.86, T84.81, I80.1, I80.2, 451.83
PE	I26
VTE	I26, I82, I80.1, I80.2, 451.83, T84.86, T84.81
Hospitalization	1013699
Postoperative hemarthrosis	M25.01, M25.02, M25.05, M25.06, M25.071, M25.072, M25.073
